# Supplementary material for: Neural network-based ensemble approach for multi-view facial expression recognition
Source: PLoS One. 2025 Mar 19;20(3):e0316562. doi: 10.1371/journal.pone.0316562 (PMC11922251; doi:10.1371/journal.pone.0316562)
Supplement: Attachment — Submitted filename: Review Response.docx [file pone.0316562.s003.docx]

**Reviewer 1**

**Comment 1:** Try to put some digitizer results of the most important in this paper in the abstract. The abstract is the first image of a reader that can catch their eye or not.

**Response:** Thank you for the suggestion to include digitizer results in the abstract. We have revised the abstract to highlight key findings.

**Comment 2:** According to Ekman, the facial region around the eyes and mouth contains more information about units as compare to other regions of face." I recommend authors to add reference to this sentence.

**Response:** Thank you to highlight the importance of the reference to support the statement about the facial region around the eyes and mouth contain more information about facial expression, as per Ekman's research. We have added the appropriate reference of Ekman's article to substantiate this claim in the manuscript.

**Comment 3:** Check the entire paper for abbreviations. For example, What is LBP, authors must write full name first and use the abbreviated term when it appeared next time.

**Response:** Thank you to point out the need for consistency to define the abbreviations throughout the paper. We have reviewed the manuscript and ensured that all abbreviations, including LBP, are fully defined at their first appearance and used consistently thereafter.

**Comment 4:** Text inside the Figures is not clear. Kindly redraw or adjust the figures to maintain the quality.

**Response:** Thank you to bring our attention towards the clarity of text inside the figures. We have redrawn and adjusted Figure 3, Figure 4, Figure 5, Figure 6, Figure 7, and Figure 8 to enhance the quality, now all the text is clear and legible.

**Comment 5:** Some of the relevant works on FER, and its applications in various areas are missing from the introduction, discussions, and references. The authors should add more relevant works. some of them are suggested below:

**Response:** Thank you for the valuable feedback to include the relevant works on Facial Expression Recognition and its applications. We have updated the literature review section and discussed the latest research on facial expression recognition. Also, we have compared our proposed approach with the latest research in facial expression recognition and included the comparison in Table 8.

**Comment 6:** To check the generalizability authors must evaluate with another dataset (in-the-wild dataset) like RAFDB or FER2013.

**Response:** Thank you for your suggestion to evaluate the proposed technique on in-the-wild datasets like RAFDB or FER2013 to assess generalizability of the technique. The primary focus of this research is on the development of a pose-aware facial expression recognition technique with the K nearest neighbor for pose detection and a neural network-based extended stacking ensemble model for classification of pose-aware facial expressions. The proposed methodology was specifically designed and optimized for the Radboud faces database with multiple views. While we understand the importance of cross-dataset evaluation, our approach aims to demonstrate the efficacy and advancement within the context of the Radboud faces database, to achieve significant performance compared to state-of-the-art technique while considering the pose aware constraint.

**Comment 7:** It is observed that authors are not compared their proposed approach with recent FER studies. Suggesting authors to compare the performance of recent existing methods (Some approaches are suggested in comment no 5).

**Response:** Thank you to point out the importance of comparison of proposed approach with recent Facial Expression Recognition studies. We have conducted a performance comparison of proposed technique with the KDEF dataset to present its effectiveness. However, comparison with other datasets presents challenges due to the pose variations inherent in our methodology. We believe that our study's primary contribution lies in addressing pose-aware facial expression recognition, and the KDEF dataset serves as a relevant benchmark for this purpose.

**Reviewer 2**

**Comment 1:** The manuscript lacks novelty. The authors have presented a study that lacks motivation. Why is there a need for a new method when we can achieve more accurate results (>90%) using models developed a decade ago? They have not justified its necessity.

**Response:** Thank you for raising concerns regarding the novelty and motivation of this study. While it may be true that models developed a decade ago can achieve high accuracy, our study aims to address specific challenges in pose-aware facial expression recognition. Our proposed technique extends the stacking ensemble model to incorporate pose detection, which is a significant advancement in adapting facial expression recognition to pose variations. The novelty of this study is the ability to effectively handle pose variations, that enhance the robustness and applicability of facial expression recognition systems. We believe that this approach fills a gap in the literature regarding pose variation. The proposed techniques provide a specialized solution to pose-aware facial expression recognition, thereby justify the development of our new method.

**Comment 2:** Moreover, the overall presentation needs improvement. The weakest section is the results, which require significant enhancement. Why PCA? More advanced versions are now available. These are the questions that need to be addressed.

**Response:** Thank you for your feedback on the choice of feature extraction techniques in this research. We employed PCA and HOG feature extraction techniques independently to compare the effectiveness of two different techniques that capture different aspects of facial expressions and poses. The reason behind using the PCA is that it is an established technique for feature reduction and has proven to be one of the best performing methods for facial expression recognition, because it focuses on global variations in facial features. On the other hand, HOG features capture the local texture and shape information, these property makes these techniques suitable to detect detail of facial features and poses. In this study on pose-aware facial expression recognition, we evaluated the effectiveness of both feature extraction techniques (PCA and HOG) features separately to assess their individual contributions to the recognition performance. This approach allowed us to understand the strengths and limitations of each feature extraction technique and determine their suitability for pose aware facial expression recognition.
