# Supplementary material for: Neural network-based ensemble approach for multi-view facial expression recognition
Source: PLoS One. 2025 Mar 19;20(3):e0316562. doi: 10.1371/journal.pone.0316562 (PMC11922251; doi:10.1371/journal.pone.0316562)
Supplement: Attachment — Submitted filename: suggestions.docx [file pone.0316562.s002.docx]

1) Abbreviations not mentioned

2) A few data set reference images to be highlighted with respect to pose angles and expressions

3) Highlighting the intermediate results is required

4) Why only accuracy is considered as measuring parameter? The other measuring metric can also be applied

5) Intensive survey to be done on recent advancement methods

6) How is your work enhanced	compared to the recent advanced methods

7) Mention the discrimination conditions to identify the features
