# Supplementary material for: Neural network-based ensemble approach for multi-view facial expression recognition
Source: PLoS One. 2025 Mar 19;20(3):e0316562. doi: 10.1371/journal.pone.0316562 (PMC11922251; doi:10.1371/journal.pone.0316562)
Supplement: Attachment — Submitted filename: suggestions Response.docx [file pone.0316562.s004.docx]

**Comment 1:** Abbreviations not mentioned

**Response:** All abbreviations added in the article

a. Histogram of oriented gradient (HOG)
b. Principal component analysis (PCA)
c. Stacked neural network ensemble (SNNE)
d. Naive Bayes (NB)
e. K nearest neighbour (KNN)
f. Support vector machine (SVM)
g. Local binary pattern (LBP)
h. Scale invariant feature transformation (SIFT)
i. Radboud faces database (RaFD)
j. Binary neural networks (BNNs)

**Comment 2:** A few data set reference images to be highlighted with respect to pose angles and expressions.

**Response:** Please See Figure 2 that presents the images of all pose angles and facial expressions.

**Comment 3:** Highlighting the intermediate results is required

**Response:** Please see Table 1 which presents the intermediate results.

**Comment 4:**Why only accuracy is considered as measuring parameter? The other measuring metric can also be applied.

**Response:** Thank you for your insightful comment on evaluation metrics used in our study. We have chosen to use accuracy and the confusion matrix as the primary measuring metric because accuracy is a straightforward and widely understood metric that provides an immediate sense of the model's overall performance. The confusion matrix complements this and offers detailed insights into the classification outcomes, such as true positives, false positives, true negatives, and false negatives. Moreover, the primary goal of our study is to assess the overall correctness of our model's predictions, for which accuracy is a direct measure. The confusion matrix helps us understand the specific areas where the model performs well and where it require improvement. The other measures directly or indirectly related to accuracy and confusion matrix. We believe that accuracy and confusion matrix are the metrics, it also helpful for easier comparison and validation of our results against existing work. We also acknowledge the value of other metrics such as precision, recall, F1-score, and AUC. However, for the scope and focus of this study, we believe that accuracy and the confusion matrix sufficiently capture the performance characteristics of our model. We appreciate your suggestion and will consider it for future work where a more nuanced analysis of model performance required.

**Comment 5:** Intensive survey to be done on recent advancement methods

**Response:** Thank you for your suggestion regarding the need for an intensive survey on recent advancement methods. While we recognize the value of such a comprehensive survey, it is important to note that our study aims to present a method with lower computational costs compared to advanced deep learning approaches. The primary objective of our research is to demonstrate the feasibility and efficiency of our proposed method rather than to provide a direct comparison with computationally intensive techniques. Conducting a thorough survey on recent advancements would necessitate a separate review article, which is beyond the scope of this study. We have considered advanced techniques that are presented in [41, 42, 43, 44, 45]. Nonetheless, we appreciate your suggestion and acknowledge the importance of staying updated with the latest developments in the field.

**Comment 6:** How is your work enhanced compared to the recent advanced methods

**Response:** Thank you for your question about the enhancement of our work as compare to recent advanced methods. Our research presents a novel pose-aware facial expression recognition technique that distinguishes itself through the innovative use of an extended stacking ensemble model. Unlike other advanced methods that often involve high computational costs, our approach achieves a significant balance between accuracy and computational efficiency. We employed K nearest neighbour for pose detection and a three-level ensemble model for classification, we ensure robust performance across various facial poses. The binary neural networks at the base and meta levels, combined using probability distribution, create a strong ensemble learning framework that effectively captures the nuances of multi-pose facial expressions. The final prediction with the Naive Bayes classifier further refines the classification process. Our experimental results, demonstrates 90% accuracy with Eigen features and 160 stacked neural network ensembles, highlight the efficacy of our method. This performance is noteworthy as it matches or surpasses state-of-the-art techniques while maintains the lower computational demands, make our approach highly practical for real-world applications.

**Comment 7:** Mention the discrimination conditions to identify the features

**Response:** Thank you for your comments on discrimination conditions to identify the features. In our study, we utilized Principal Component Analysis (PCA) and Histogram of Oriented Gradients (HOG) to extract discriminative features for pose-aware facial expression recognition. PCA was employed to reduce the dimensionality of the feature space and preserve the most significant features. We selected 50 most prominent features, it was decided by varying the principal components, and the best performing number of features selected. It allow us to highlight the most informative features for classification. HOG was used to capture the essential structural and gradient information of the facial images, which is crucial to distinguish between different facial expressions. These methods were chosen due to the effectiveness of the feature extraction methods to highlight relevant features and reduce computational complexity, It ensures that the model remains efficient and accurate.
